# Tutorial in oral antithrombotic therapy: Biology and dental implications

**DOI:** 10.4317/medoral.19140

**Published:** 2013-03-25

**Authors:** Hamid R. Fakhri, Sok J. Janket, Elizabeth A. Jackson, Alison E. Baird, Richard Dinnocenzo, Jukka H. Meurman

**Affiliations:** 1 DDS, Research Associate, General Dentistry, Boston University, Henry M. Goldman School of Dental Medicine, U.S.A; 2 DMD, MPH, Research Associate professor, General Dentistry, Boston University, Henry M. Goldman School of Dental Medicine, U.S.A; 3 MD, MPH, Assistant professor, Preventive Cardiology, Internal Medicine, University of Michigan, Ann Arbor, Michigan, U.S.A; 4 MD, MPH, Professor, Neurological disorders and Stroke, SUNY Downstate Medical Center, Brooklyn, NY, USA; 5 DMD,MD, Clinical Associate Professor, Oral and Maxillofacial surgery, Boston University, Henry M. Goldman School of Dental Medicine, Boston, MA, U.S.A; 6 DDS, MD, Professor, Department of Oral and Maxillofacial Diseases, Helsinki University Central Hospital, and Institute of Dentistry, University of Helsinki, Helsinki, Finland

## Abstract

Objectives: Recent developments of new direct oral anticoagulants that target specific clotting factors necessitate understanding of coagulation biology. The objective of this tutorial is to offer dental professionals a review of coagulation mechanisms and the pharmacodynamics of the conventional and new oral anticoagulants. Also, we summarized the dental implications of the conventional and new anticoagulants. Method: We searched Medline using search terms “antithrombotic”, “antihemostasis” or “anticoagulation” and combined them with the search results of “dental”, “oral surgery” or “periodontal”. We restricted the results to “human” and “English”. Results: The early coagulation cascade, the new cell-based coagulation model, the pharmacokinetics and pharmacodynamics of conventional antithrombotics, and new oral anticoagulants were reviewed. The new direct factor Xa inhibitors and the direct thrombin inhibitor (s), called direct oral anticoagulants (DOAs) have rapid onset of action, fast elimination on cessation, and fewer drug-drug or drug-food interactions than warfarin. However, the lack of antidotes raises concerns that some dental procedures may trigger serious hemorrhagic events. Additionally, careful perioperative withdrawal and resumption protocols for the DOAs are reviewed, because DOAs’ blood levels are dependent on renal function. Also, various reversal strategies in the event of excessive bleedings are summarized. Perioperative management of dental patients taking new DOAs and conventional oral anticoagulants are also discussed. However, the perioperative strategies for DOAs are yet to be validated in randomized trials.

** Key words:**Coagulation cascade, cell-based coagulation model, factor Xa inhibitors, direct thrombin inhibitors, prothrombin complex concentrates.

## Introduction

The increasing elderly population and long life-expectancy lead to a high prevalence of chronic illnesses including heart disease and stroke. (1) These diseases often require antithrombotic therapy to prevent thromboembolic (TE) events. The indications for antithrombotic therapy are to prevent TE events and stroke in: (I) Atrial fibrillation and other cardiac arrhythmias; (II) Venous thromboembolism (deep vein thrombosis, pulmonary embolism); (III) Acute coronary syndrome and myocardial infarction; (IV) Pulmonary hypertension; and (V) Cardiac valve disease and prosthetic valve replacement. (2,3) 

Oral antithrombotic drugs can be divided into two categories: anti-platelets and anticoagulants.  Table 1 summarizes these categories. Acetylsalicylic acid (aspirin) is the most widely used antiplatelet agent and the most commonly prescribed oral anticoagulant has been warfarin. Consequently, instructional articles automatically refer to oral anticoagulants as warfarin and its derivatives. (2,4-7) However, the coagulation concept has been modified into a new, cell-based hemostasis model and several new oral anticoagulants targeting specific clotting factors have been introduced in 2010 – 2011. Only recently, two cursory reviews on these new direct oral anticoagulants (DOAs) have appeared in the dental literature (8,9). The objectives of the present review are (1) to educate general dental professionals about coagulation cascade and the pharmacology of new and old anticoagulants and (2) to suggest peri-surgical management strategies for patients taking new DOAs. Concurrently, we call for more research action utilizing these new DOAs in dental setting.

**Table 1:**
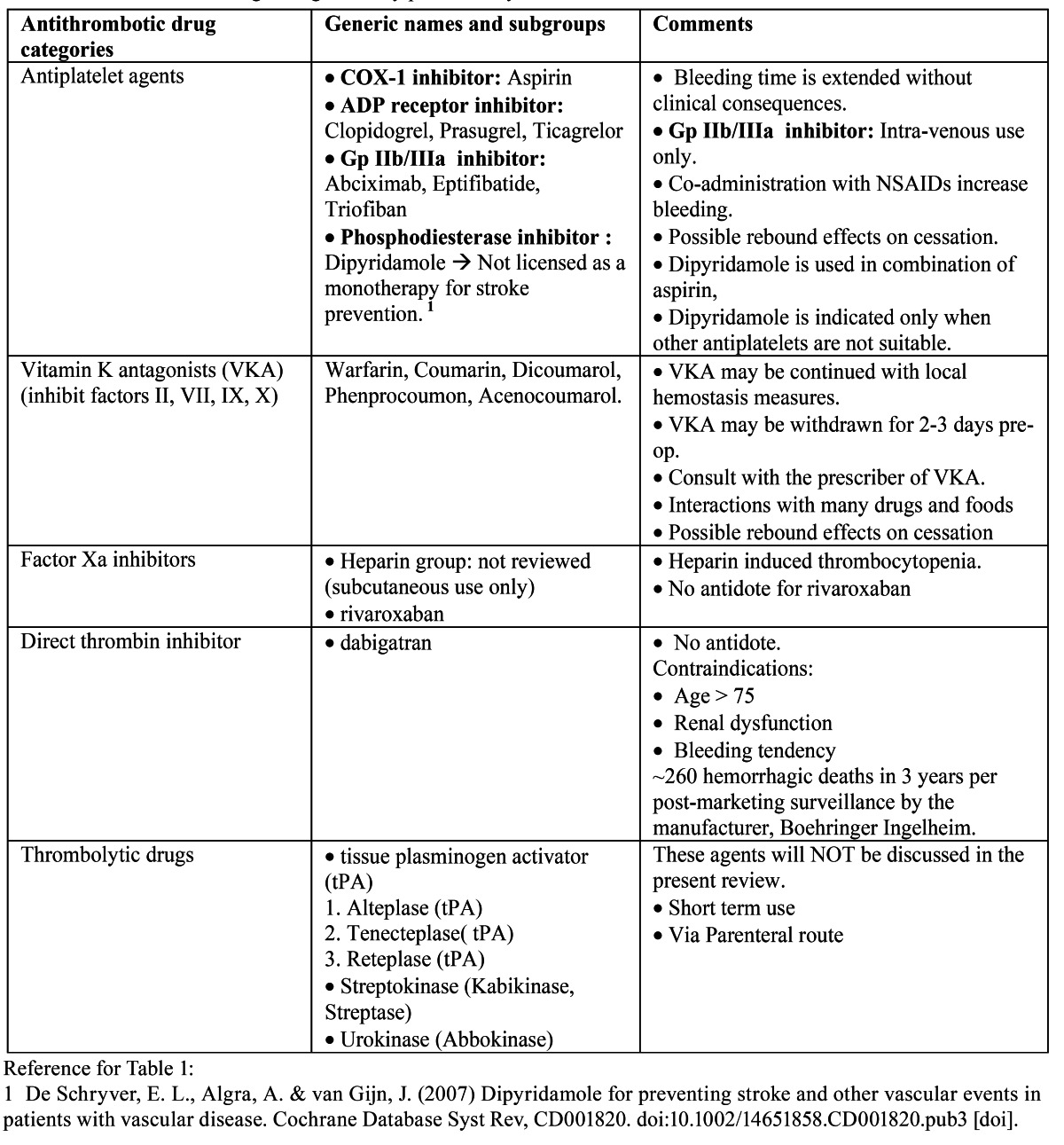
Antithrombotic drugs categorized by pharmacodynamics.

To conduct this review, we searched PubMed with search terms “anti-platelet”, “antithrombotic”, “anticoagulation”, or “anti-hemostasis”, published between 1966- 2012 and in a separate search, we used the search terms “dental” “oral surgery” or “periodontal” and merged two searches. We collected 113 dentistry-related references. In the first section of this review, we reviewed the early coagulation cascade; in the second section, we introduced the new coagulation model; in the third section, we presented the new direct oral anticoagulants; and in the fourth section, we discussed perioperative management strategy.

## Concepts on early coagulation cascade

Hemostasis involves a multipart physiological process that limits blood loss at the site of an injury while maintaining normal blood flow elsewhere in the circulation.

An early model of coagulation derived from in vitro experiments and presented in the mid-1960s (10,11) involved a series of biological steps via intrinsic and extrinsic pathways leading to a common pathway to activate factor X (f.X). The intrinsic pathway includes factors XII (f.XII), XI (f.XI), IX (f. IX) and VIII (f.VIII) as well as prekallikrein and kininogen. The extrinsic path-way is composed of factor VII (f.VII) and tissue factor (TF).

In the intrinsic pathway, activated f.XII leads to activated f. IX (f. IXa) after several steps of molecular activation. In turn, f. IXa converts f.X to activated f.X (f.Xa). On a collateral, extrinsic pathway, f.X can be activated by f.VII and tissue factor (TF/f.VII complex). Thus, f.Xa from both pathways converges in a common pathway whose constituents are factors V (f.V), prothrombin (f.II), fibrinogen (f.I) and thrombin.

**Figure 1:**
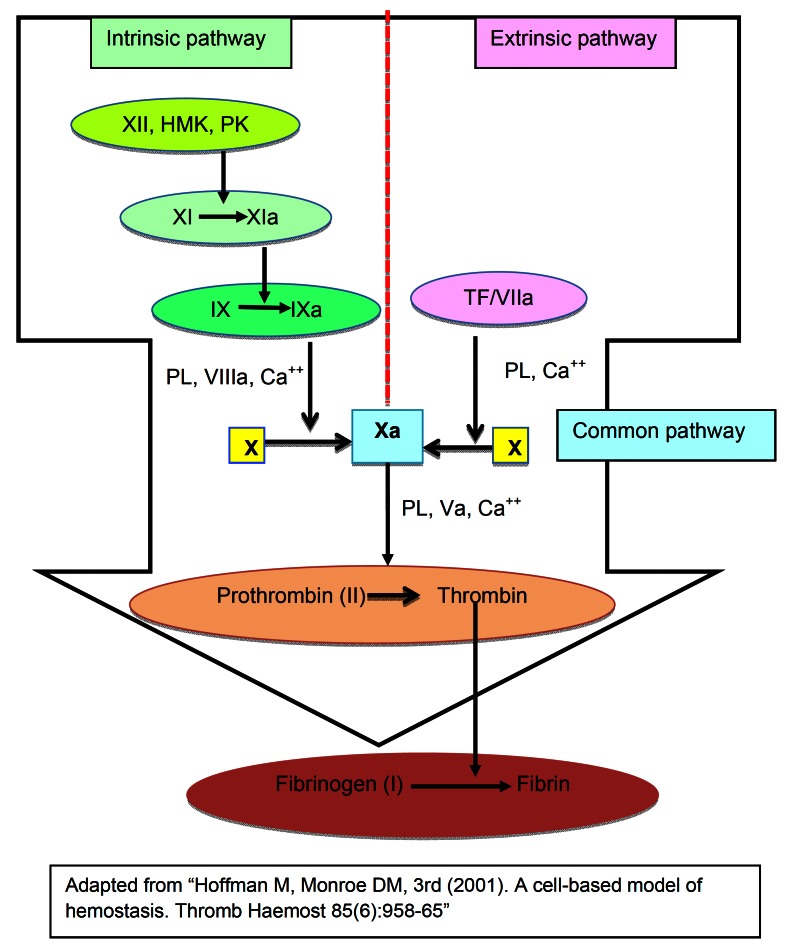
Early coagulation cascade.

Either way, activated f.X (f.Xa) is the key player in converting prothrombin (f.II) to thrombin, which converts fibrinogen (f.I) to fibrin. The activated partial thromboplastin time (aPTT) is a laboratory test for intrinsic pathway function and prothrombin time (PT) test assesses the function of the extrinsic pathway. (12) Each clotting factor leads to the activation of another, leading to a sufficient thrombin generation necessary for the formation of fibrin. This sequence of events is summarized in figure 1. Within this cascade model, the involvement of intrinsic and extrinsic pathways is considered independent. (13) 

While this concept was a huge achievement in the understanding of molecular interactions regulating coagulation, it had several limitations in explaining the hemostatic process in vivo. Thus, several new theories were proposed: one that extrinsic and intrinsic pathways are actually working inter-dependently has been well-received. (12) An example of this collaboration of two pathways is that the f.VIIa/TF complex from the extrinsic pathway can activate f.IX in the intrinsic pathway as well as f.X in the common pathway. (14) These concepts generated a new paradigm advocating that cellular components are the key players in coagulation which is controlled by the expression of a variety of cell features and protein receptors that localize the components of coagulation on specific cell surfaces. Thus, a concept of coagulation evolved from a mechanism being controlled by kinetics of various proteins to a new concept of cellular components controlling the coagulation process. (12) We now review the new, cell-based coagulation model.

## Concepts on the new, cell-based coagulation model

The new model of coagulation is a well-coordinated series of cell activation, culminating in a blood clot. (12) Three stages proposed in this new coagulation models are initiation, amplification (priming) and propagation. In the initiation stage, TF from fibroblasts (or other TF bearing cells such as monocytes or endothelial cells) binds the activated factor VII, (a circulating coagulation factor) and forms the TF/VIIa complex. TF/VIIa complex in turn activates factors IX and X. The activated factor X (Xa) forms a Xa / Va complex on the surface of fibroblasts and generates a sufficient amount of thrombin to induce platelet activation. (15) Thus, coagulation now moves to a more efficient site, the negatively charged, platelet surface.

In the second stage, the amplification (or priming) phase, the coagulation now has moved from tissue factor bearing fibroblasts to platelets that are much better suited for the coagulation with their negatively charged surfaces. Platelets adhere and accumulate cofactors on their surface and amplify the coagulation. Platelets and coagulation factors are activated by thrombin, which in turn binds and cleaves platelet protease-activated receptors (PAR1 and PAR4), triggering a complex signaling cascade. Furthermore, thrombin activates factors V, VIII, and XI also. (15) This is a crucial point many health care professionals are concerned about, because multifaceted anticoagulation ability will complicate the reversal process if excessive hemorrhage occurs. (16) 

**Figure 2:**
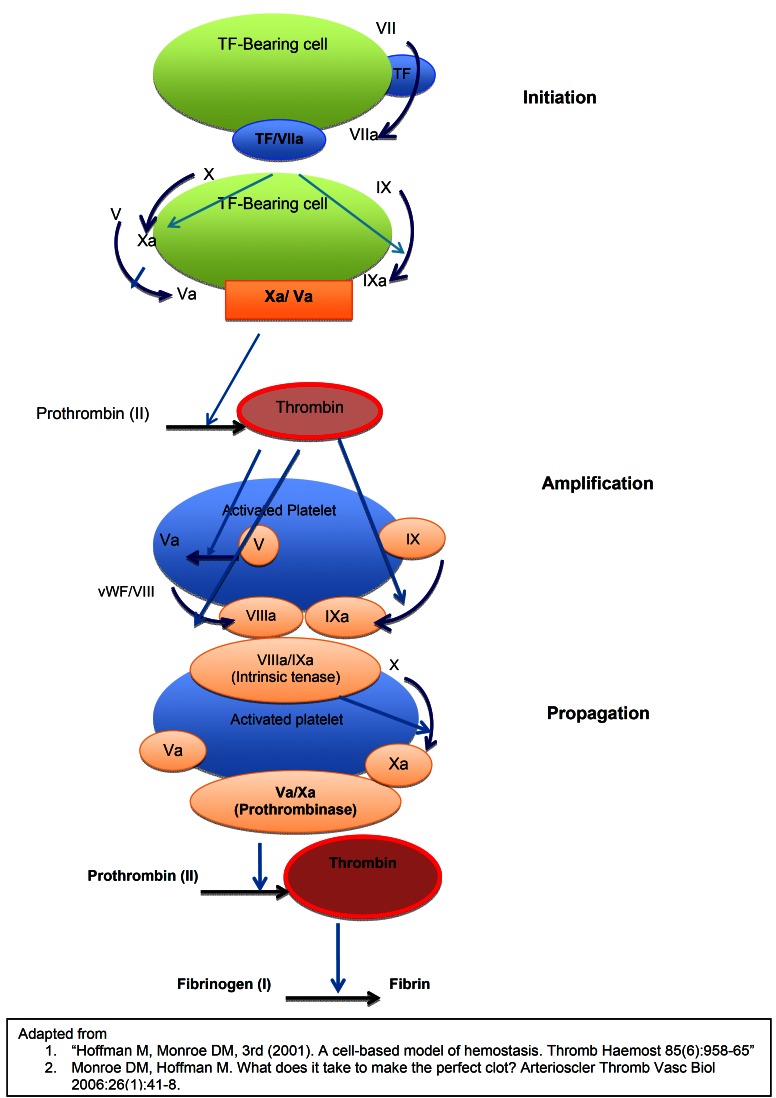
New cell-based coagulation model.

In the final propagation phase, the tenase (factor “ten” activator) and prothrombinase (f.Xa / f.Va) complexes are assembled on the platelet surface, and large-scale thrombin generation takes place. (12) Tenase can originate from either the extrinsic pathway or from the intrinsic pathway. Intrinsic tenase is made of f.IXa and f. VIIIa with Ca++ and extrinsic tenase consists of TF/f.VIIa with Ca++. Intrinsic tenase is 50 times more efficient than extrinsic tenase in f.Xa expression. Additionally, prothrombinase complex (f.Xa / f.Va) is protected from tissue factor inhibitors, for it does not contain TF and thus a powerful production of f.Xa and thrombin formation results. This leads to a large scale fibrin formation. (15) These progressive phases are summarized in figure 2.

Under normal conditions, tissue factors do not come into contact with circulating vascular clotting factors. However, in some instances such as percutaneous coronary intervention, hemodialysis, or cardiac valve replacement surgery, these two components come into contact resulting in a clot and subsequent adverse outcomes, such as myocardial infarction or stroke. In various inflammatory states, TF expression can also be up-regulated in monocytes and endothelial cells by bacterial antigens, inflammatory cytokines, (17) or tumor necrosis factor. (18) This explains the hyper-coagulative state accompanying infection. Now that we have reviewed both old and new coagulation models, it will be easier to understand where the new antithrombotic drugs inhibit the coagulation mechanism.

## New antithrombotic agents

3-1. New antiplatelet drugs 

Platelets play a central role in thromboembolic (TE) events and four potential receptors have been recognized in platelet activation. (19) 

The first pathway via the thromboxane A2 synthesis is through which the conventional antiplatelet agent, aspirin, exerts its antiplatelet action. Aspirin is a cyclo-oxygenase (COX)-1 inhibitor that irreversibly inhibits platelet activation. It is an efficient and inexpensive drug but cause mucosal irritation and may cause internal bleeding. Thus, in recent years, a group of new antiplatelet agents has been developed.

**Figure 3:**
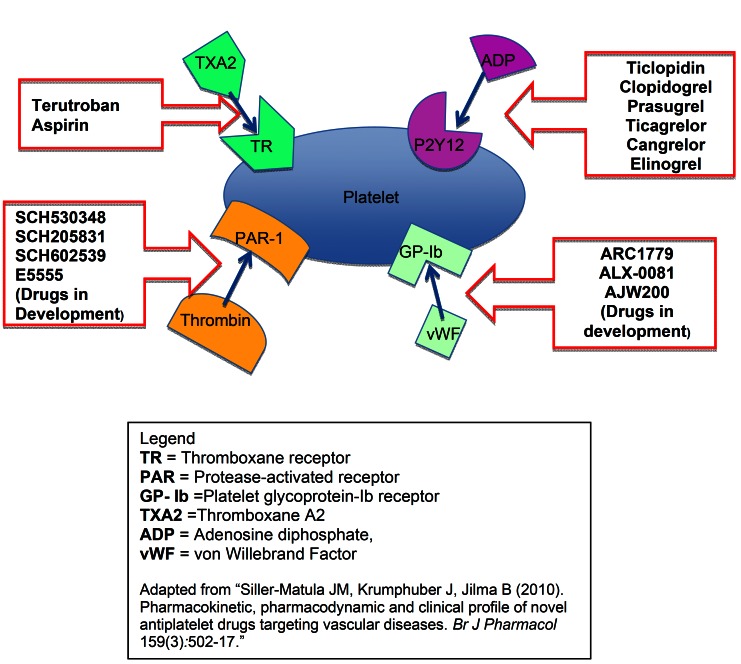
Pathways of antiplatelet mode of action.

The second pathway includes thienopyridines that include clopidogrel (Plavix®), and prasugrel (Effient®) that irreversibly block adenosine diphosphate (ADP) activation of platelets through the ADP receptor P2Y12. (20) A non-thienopyridine P2Y12 receptor inhibitor, ticagrelor (Ticlid®) is a reversible anti-platelet agent much more potent and has faster onset of action than clopidogrel. However, it accompanies more adverse effects such as dyspnea, hematoma and excessive bleeding. (21) The third pathway is via von Willebrand factor (vWF) and platelets’ glycoprotein (Gp)-Ib receptor. When rupture of an atherosclerotic plaque exposes collagen and vWF, the A1 domain of vWF binds to the Gp-Ib receptor of platelet. The fourth pathway for platelet activation is via protease activator receptor-1 (PAR-1). If platelet activation is blocked through any one of these pathways, platelet membrane glycoprotein IIa/IIIb cannot bind to fibrinogen and unable to generate a hemostatic plug. (22) These four pathways through which platelet aggregation can be inhibited are illustrated in figure 3.

3-2. New direct oral anticoagulants (DOAs)

The conventional vitamin K antagonist (VKA), warfarin, has several drawbacks that limit its clinical utility: numerous drug-drug and drug-food interactions, the genetic differences in VKA metabolism via cytochrome P450, and wide inter-individual differences in pharmacokinetics as well as the narrow therapeutic window which requires frequent testing. Therefore, patients’ compliance directly affects its clinical efficacy. Warfarin stays in the therapeutic range approximately 60% of the time, with an average of ~12% of the time in the supra- and ~20% of the time in the sub-therapeutic range. (23) For these reasons, the impetus for development of safer anticoagulants has been active resulting in several new direct oral anticoagulants (DOAs). These are direct factor Xa inhibitors rivaroxaban (Xarelto®), apixaban (Eliquis®), edoxaban and direct thrombin inhibitor dabigatran (Pradaxa®). ( Table 2, Table 3) summarizes the pharmacokinetics pharmacodynamics, including dietary potentiators or inhibitors of new DOAs as well as warfarin.

**Table 2:**
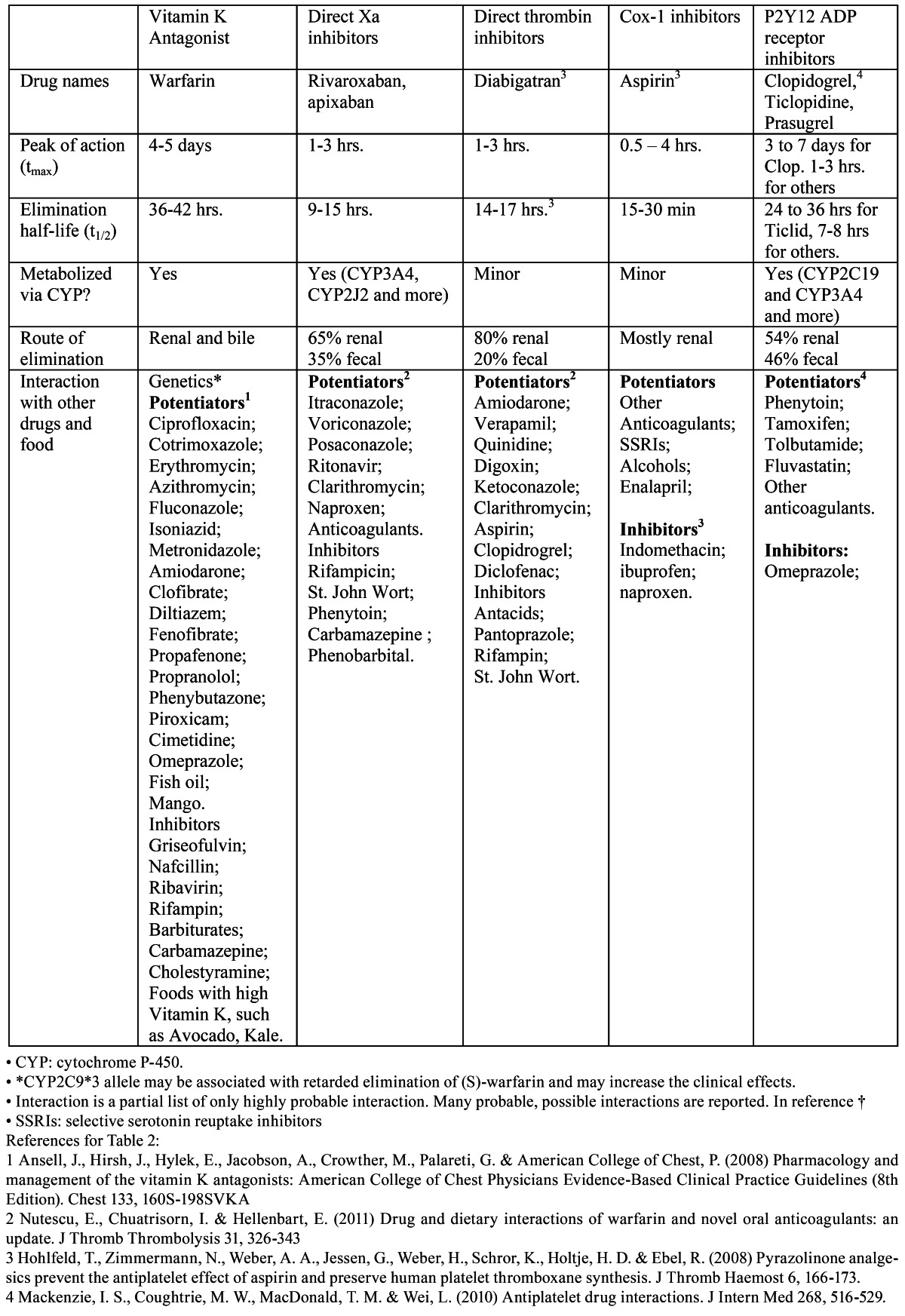
Pharmacokinetics of warfarin and new direct oral anticoagulants.

**Table 3:**
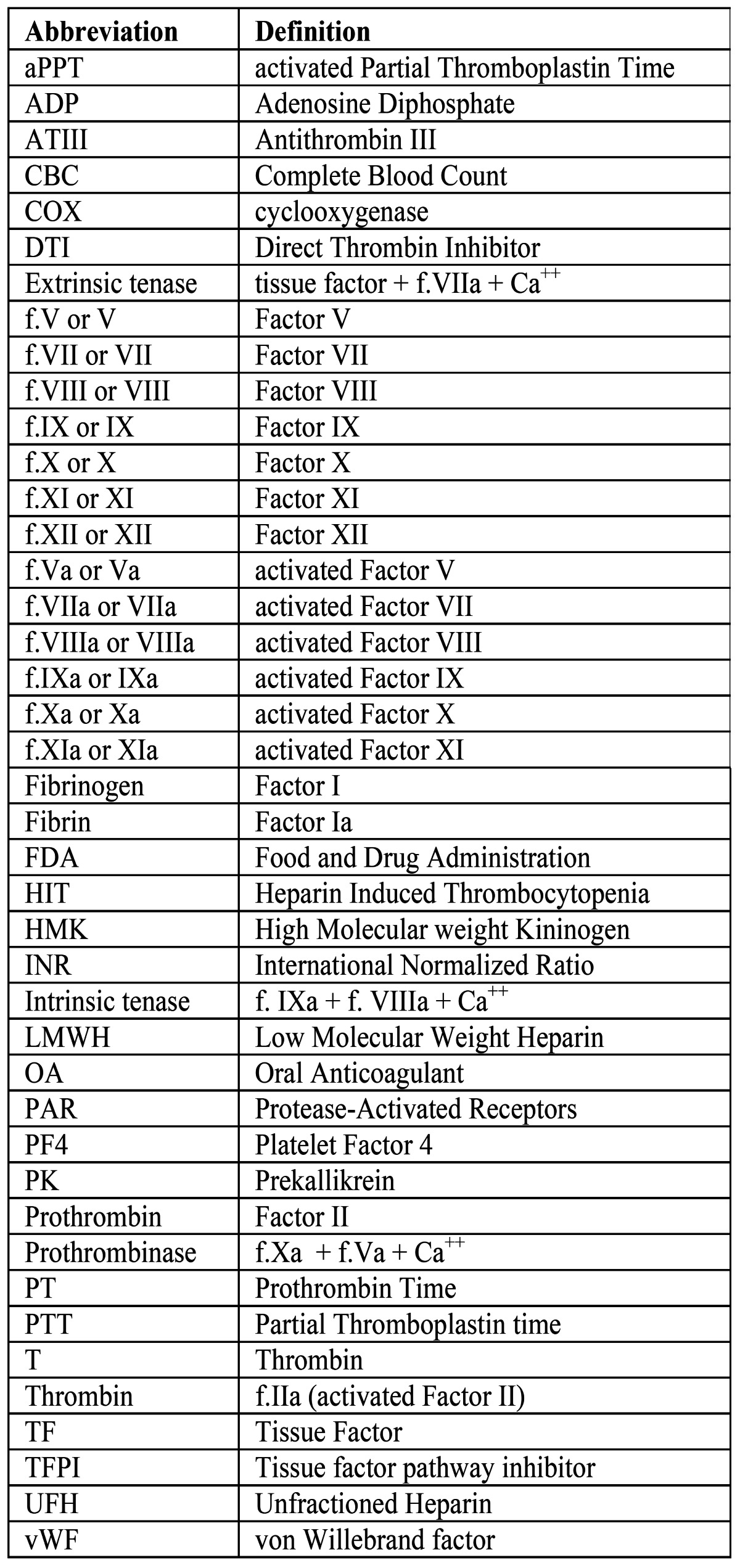
Table of abbreviations.

New DOAs have rapid onset of action, fast elimination on cessation and have fewer drug-drug or drug-food interactions. This obviates the need for frequent testing as with warfarin. However, no reversal agents are available at present and minimal trauma such as subgingival periodontal scaling, gingivectomy, or simple exodontia have potential to become serious hemorrhagic events. We will discuss the new direct oral anticoagulants in detail.

3-2-a. Direct factor Xa inhibitors (Xaban)

Rivaroxaban is one of the direct factor Xa (f.Xa) inhibitors approved by FDA for the prevention of thromboembolic (TE) events in patients with atrial fibrillation.(24) The other drug in this group is apixaban for which FDA just approved its use in Jan. 2013. These drugs inhibit both free f.Xa and bound f.Xa within the prothrombinase complex. Rivaroxaban reaches its peak plasma level at ~3 hours after administration and ~65% is excreted via urine and ~35% via biliary and fecal routes. Therefore, in the elderly or in persons with renal impairment, rivaroxaban would have a prolonged half-life.

3-2-b. Direct thrombin inhibitor (DTI)

Dabigatran is one of the direct thrombin inhibitors (cf. to Figs. 1,2) approved by FDA for stroke prevention in non-valvular atrial fibrillation (A-fib). (25) It has a half-life of 14 -17 hours and 80% is excreted through urine and 20% through the fecal route. Therefore, in persons with impaired renal function, the half-life will increase and invasive procedures that require cessation of dabigatran should be determined according to renal function. (26) Although reliable pharmacodynamics and pharmacokinetics eliminate frequent testing and ecarin clotting time (ECT) and thrombin clotting time (TT) assays may assess antithrombotic action of new DOAs, there is no dependable way of determining therapeutic plasma levels of dabigatran. (27) Neither aPTT, PT nor INR is sufficiently precise to measure the clinically effective serum level of DOAs. (28) 

Not as extensive as warfarin, some drug-drug interactions have been demonstrated with dabigatran: with quinidine, ketoconazole, amiodarone, and verapamil that can increase the dabigatran plasma concentrations. (26) Co-administration of other anticoagulants or non-steroidal anti-inflammatory drugs will also increase dabigatran level. On the other hand, rifampicin and some proton pump inhibitors such as omeprazole will decrease dabigatran levels.

## Clinical implications of the new anticoagulants

Currently, there is no published research evidence utilizing new DOAs in the dental setting. However, we summarized perioperative strategies that appeared in medical journals so that dental professionals will have adequate basic knowledge when they consult physicians regarding their patients on new DOAs. In addition, active research in the development of the antidotes for new DOAs makes it imperative for dental professionals to have a clear understanding of coagulation mechanisms.

4-1. General dental considerations

Scheduling early in the week and early morning of the day appointment may be necessary to afford additional visit in case of excessive bleeding. Furthermore, additional supplies to deal with the post-surgical bleeding should be considered for patients taking anticoagulants. (4,29,30) Some suggested additional supplies are absorbable hemostatic dressings such as oxidised cellu-lose (Surgicel®), collagen sponge (Haemocollagen®) or resorbable gelatin sponge (Spongostan®), along with resorbable and non- resorbable sutures. Also suturing the extraction sites and pressure application with gauzes saturated with tranexamic acid has also been suggested to prevent post-surgical bleeding. (31) 

Careful preoperative assessment of patients’ medical profile was a common recommendations and some suggested routine laboratory studies including: a complete blood count (CBC), prothrombin time (PT), activated partial thromboplastin time (aPTT), INR in case of VKA, and bleeding time. Arranging an after-hours response team should be considered for those general dental practitioners (GDPs) without hospital privileges to counter postoperative bleeding. Most bleeding risk studies compared those who stopped warfarin to those who stayed on warfarin during the dental procedures and found that bleeding was not significantly different. However, if the bleeding incidence was compared to the controls not taking warfarin, the indirectly calculated relative bleeding risk was about a 10-fold increase in those who are on warfarin (9-10%) than those who do not take anticoagulants (1%). (32) In addition, some delayed bleedings were reported after 24 or 48 hours.(33) Therefore, post-surgical bleeding in patients taking anticoagulants is much greater than what is expected in the general dentist’s office (GDPs) and setting up an effective post-surgical response plan will be necessary.

As to the risk factors for post-dental surgical bleeding, we postulated that (a) the severity of systemic morbidity requiring a high degree of anticoagulation, (b) comorbidity such as renal insufficiency and hepatic dysfunction, and (c) the surgical trauma involved in dental procedures may all influence the bleeding risk. However, there is no clear definition of significant bleeding in the dental setting or clear guidelines for dental procedures with high bleeding risk. We reviewed the definition of significant bleeding by several authors and found Lockhart and colleague’s definition is the most reasonable. (34) They defined significant bleeding as.

1) Continues beyond 12 hours; (However,) in our opinion, even bleeding that continues to 5-6 hours should be considered significant.

2) Causes the patient to call, return to the dental practitioner, or visit the emergency department;

3) Results in the development of a large hematoma or ecchymosis within the oral or adjacent soft tissues;

4) Requires a blood transfusion or other blood products.

As for the determination of invasiveness of dental procedures, Muthukrishnan et al. surveyed general dental practitioners (GDPs) and found that 71% of the GDPs surveyed considered subgingival debridement as a high bleeding risk, 48% inferior alveolar block, and 32% subgingival restoration as high bleeding risks. (35) For exodontia, single and up to 3 extractions were considered a minor bleeding risk.

4-2. Managing patients on anticoagulants in dental-surgical setting

**Figure 4:**
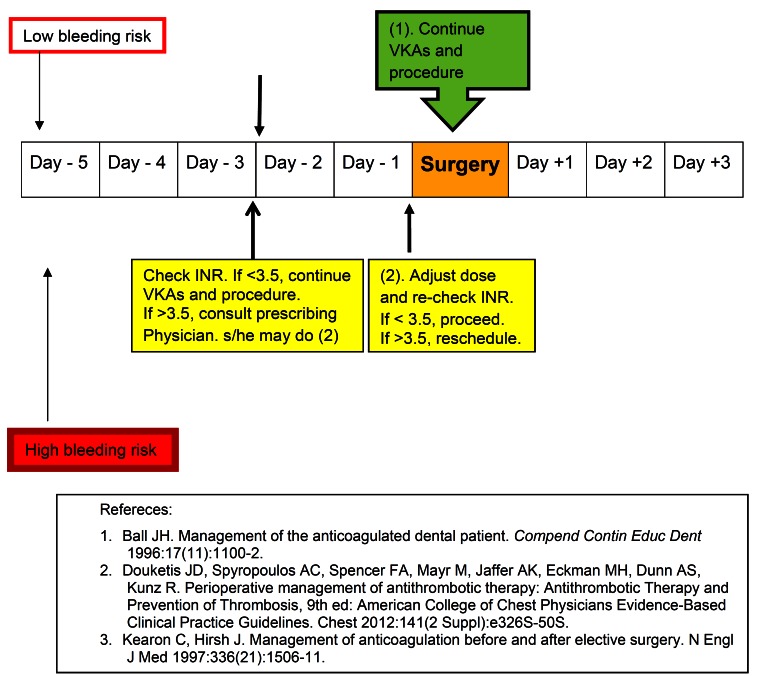
Perioperative management strategies for Vitamin K antagonists.

Recent American College of Chest Physicians’ (ACCP) Evidence-Based Clinical Practice Guidelines reflects the controversy surrounding the withdrawal/continuation of vitamin K antagonists (VKAs) and modified the past recommendation: In dental procedures with low bleeding risk, VKAs may be continued with implementation of local haemostatic measures or may be inter-rupted for 2-3 days prior to surgery. (28) However, most dental professionals favor continuing VKAs when International Normalized Ratio (INR) is below 3.5. As Ball emphasized (36) (1) consulting the prescribing physician(s) to ascertain the patient’s medical status that required anticoagulation therapy, (2) checking the latest INR and testing it again on the day of the dental procedure may be very important in preventing thromboembolic (TE) events because VKA levels fluctuate with diet and patient’s compliance. If INR > 3.5 with moderate bleeding risk, withdrawal of VKA with physician’s approval for 2-3 days may be necessary. Also hemostatic measures described in section 4-1 should be employed. In high bleeding risk procedures including periodontal surgery, surgical extraction, multiple extractions (> 3 teeth), osteoplasty, and extensive head and neck surgery, a withdrawal of VKA in conjunction with subcutaneous or intravenous heparin usage was recommended. (36) This protocol is illustrated in figure 4.

**Figure 5:**
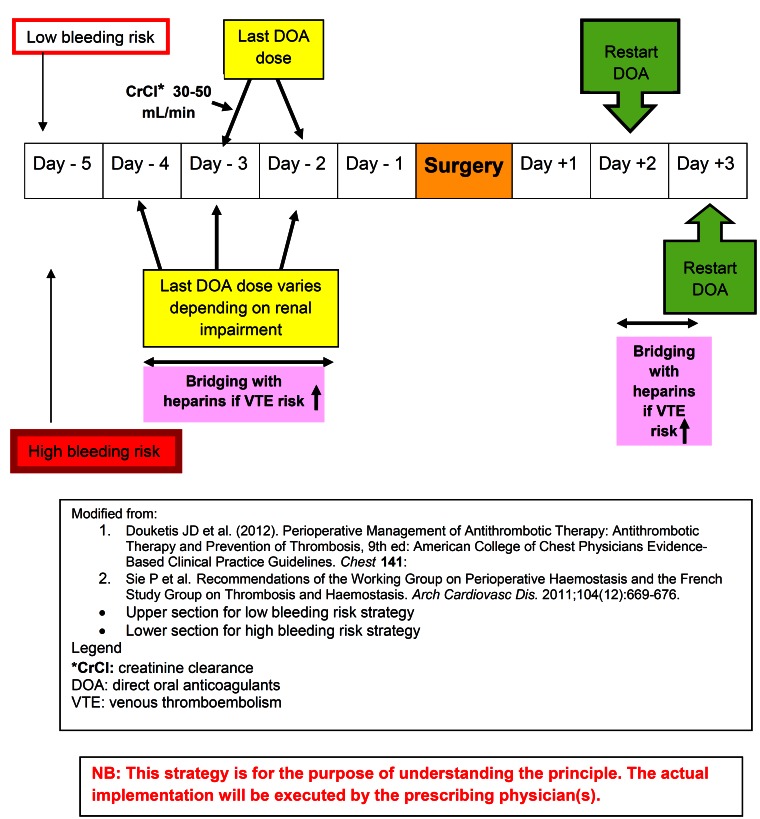
Perioperative management strategies for Direct Oral Anticoagulants.

The withdrawal of new DOAs protocol is more complex than VKA because no simple reversal agents are available. The protocol is governed by the half-life of the drug, invasiveness of the (dental) procedures, the patients’ co-morbidities, and TE risks. As the elimination half-life of dabigatran is 12-18 hours, if the bleeding risk is high, the last dose should be given 3 days prior to the surgery (skip for 2 days). (28) If moderate hemostasis is acceptable, the last dose should be given -2 days (skipping 1 day). In case of moderate renal impairment, namely creatinine clearance is (CrCl) 30-50 mL/min, skip 4 doses (skip for 2 days) for low bleeding risk procedures and 6-8 doses (skip 3-4 days) for high bleeding risk procedures.(28,37) For rivaroxaban with half-life of ~9-10 hours, the renal function is less critical in drug elimination. Thus, the last dose should be given on -2 days for the low bleeding risk and on -3 days for high bleeding risk procedures respectively. Another factor that should be considered is the individual patients TE risk. If TE risk is high, bridging with heparin(s) without overlapping with DOAs is suggested. The perioperative protocols for DOAs are summarized in figure 5.

For the antiplatelet agents, the perioperative protocol is similar to the other antithrombotics. If the bleeding risk of procedure is low, not stopping the antiplatelet is recommended. (37) Resumption of aspirin results in nearly immediate maximal drug effects. However clopidogrel will take almost 7 days to reach the maximal effective level. Administering the initial loading dose may shorten this lag period (37).

4-3. Reversal of direct oral anticoagulants 

Although new DOAs have the advantage of stable pharmacological action and less interaction with foods or other drugs, the most important disadvantage of these new drugs is the lack of specific reversal agents. In case of excessive bleeding, the knowledge of reversal strategy will empower dental professionals.

In case of warfarin-related major bleeding, vitamin K may be administered. However, it takes almost 24 hours to bring INR to normal range. Therefore, in urgent hemorrhage caused by VKA overdose, nonspecific pro-hemostatic agents, such as prothrom-bin complex concentrates (PCCs), are indicated. (38)

The first line of defense in any bleeding is discontinuation of the causative medication and utilization of local measures such as pressure with tranexamic acid-soaked gauzes, or other hemostatic products. If DOA administration is recent, gastric lavage or activated charcoal can be effective. (38) If these measures fail, hemodialysis or administration of either 3-factor (II, IX, and X) or 4-factor (II, VII, IX, and X) PCC should be considered. Also in the murine model, fresh frozen plasma (FFP) was able to reduce hemorrhage. However, due to the inconvenience of thawing FFP, PCCs or recombinant factor VIIa (rFVIIa) is gaining popularity over FFP.

Ex vivo trial in humans also showed that activated four factor PCC reversed the effects of rivaroxaban and dabigatran. (39,40) In the U.S., unactivated 4- factor PCCs are not available but activated PCCs that contain similar concentrations of non-activated factors II, IX, and X and activated factor VII are available. PCC has been used in the dental setting successfully. (41) However, caution must be exercised in using PCC or rFVIIa because they carry thrombogenic risks.

## Conclusion

Although the rate of thromboembolic events is low, (approximately 0.5%) in the dental setting (42,43), most dental professionals favor continuing VKAs (warfarin) for dental surgery because the adverse outcomes of discontinuation are much more serious than bleeding risks. However, the emergence of new direct oral anticoagulants poses some challenges due to the lack of simple reversal agent in the event of post-surgical bleeding. Future randomized trials utilizing new direct oral anticoagulants like the direct factor Xa inhibitors or direct thrombin inhibitors are warranted.
